# Simultaneous Surgical Approach with Hyperthermic Intraperitoneal Chemotherapy (HIPEC) in Patients with Concurrent Peritoneal and Liver Metastases of Colon Cancer Origin

**DOI:** 10.3390/jcm12113860

**Published:** 2023-06-05

**Authors:** Rafael Morales-Soriano, Cristina Pineño-Flores, José Miguel Morón-Canis, Francisco Javier Molina-Romero, José Carlos Rodriguez-Pino, Julia Loyola-Miró, Francisco Xavier Gonzalez-Argente, Elías Palma-Zamora, Mónica Guillot-Morales, Sandra Giménez, Melchor Alvarez-Mon, Miguel A. Ortega, Juan José Segura-Sampedro

**Affiliations:** 1 Department of Digestive Surgery, University Hospital Son Espases, 07120 Palma de Mallorca, Spain; cristina.pineno@ssib.es (C.P.-F.); josem.moron@ssib.es (J.M.M.-C.); xmolina@ssib.es (F.J.M.-R.); josec.pino@ssib.es (J.C.R.-P.); julia.loyola@ssib.es (J.L.-M.); xavier.gonzalez@ssib.es (F.X.G.-A.); eliasf.palma@ssib.es (E.P.-Z.); juan.segura@ssib.es (J.J.S.-S.); 2 Faculty of Medicine, University of the Balearic Islands, 07122 Palma de Mallorca, Spain; 3 Health Research Institute of the Balearic Islands (IDISBA), 07120 Palma de Mallorca, Spain; 4 Royal Academy of Medicine of the Balearic Islands, 07120 Palma de Mallorca, Spain; 5 Department of Medical Oncology, University Hospital Son Espases, 07120 Palma de Mallorca, Spain; monicam.guillot@ssib.es (M.G.-M.); sandra.gimenez@ssib.es (S.G.); 6 Department of Medicine and Medical Specialties, Faculty of Medicine and Health Sciences (IRYCIS), University of Alcalá, 28801 Alcalá de Henares, Spain; mademons@gmail.com (M.A.-M.); miguel.angelortega92@gmail.com (M.A.O.)

**Keywords:** HIPEC, colon cancer, liver metastases, peritoneal carcinomatosis, combined resection

## Abstract

**Background**: Simultaneous liver resection and peritoneal cytoreduction with hyperthermic intraperitoneal chemotherapy (HIPEC) remains controversial today. The aim of the study was to analyze the postoperative outcomes and survival of patients with advanced metastatic colon cancer (peritoneal and/or liver metastases). **Methods:** Retrospective observational study from a prospective maintained data base. Patients who underwent a simultaneous peritoneal cytoreduction and liver resection plus HIPEC were studied. Postoperative outcomes and overall and disease free survival were analyzed. Univariate and multivariate analyses were performed. **Results:** From January 2010 to October 2022, 22 patients operated with peritoneal and liver metastasis (LR+) were compared with 87 patients operated with peritoneal metastasis alone (LR−). LR+ group presented higher serious morbidity (36.4 vs. 14.9%; p: 0.034). Postoperative mortality did not reach statistical difference. Median overall and disease free survival was similar. Peritoneal carcinomatosis index was the only predictive factor of survival. **Conclusions**: Simultaneous peritoneal and liver resection is associated with increased postoperative morbidity and hospital stay, but with similar postoperative mortality and OS and disease free survival. These results reflect the evolution of these patients, considered inoperable until recently, and justify the trend to incorporate this surgical strategy within a multimodal therapeutic plan in highly selected patients.

## 1. Introduction

In the last two decades, the appearance of new cytostatics and biological agents, together with the improvement of perioperative care and surgical technique, has changed the prognosis of metastatic colon cancer. Surgical resection of liver metastases, applied in more than 30% of patients, has achieved 5-year survival rates around 40% [1,2] and something similar has occurred with the surgical resection of isolated pulmonary metastases [2,3]. Peritoneal metastases are the metastatic form with the worst prognosis, being the second cause of death in colon cancer, probably due to the lower penetration of cytostatics in the peritoneal nodules [4]. However, some studies have reported fairly similar survival results between patients with liver metastases and those with peritoneal metastases [5,6].

It is estimated that approximately 8% of patients with colon cancer develop hepatic and peritoneal metastases simultaneously [1,7]. Simultaneous resection of the primary tumor together with liver metastases is now routinely performed [8], but until 1999, the concomitant presence of liver and peritoneal metastases was considered a contraindication for surgical treatment and these patients were considered unresectable and amenable only to palliative adjuvant chemotherapy, with an overall survival of 12 months [4,7]. Elias et al. and a consensus statement showed that the presence of three liver metastases with a low peritoneal tumor burden (PCI < 12) did not suppose an absolute contraindication for simultaneous treatment with CRC + HIPEC [9,10,11]. Since then, several studies and meta-analyses have published acceptable morbidity and mortality results, with a median survival of around 25–48 months in selected patients treated with simultaneous surgical treatment of both lesions and the administration of HIPEC and adjuvant chemotherapy [1,10,11,12,13,14,15,16,17,18,19,20,21]. Despite these publications, simultaneous resection of liver and peritoneal metastases remains controversial due to its increased morbidity, mortality, and delayed administration of adjuvant chemotherapy, and for these reasons it has not been established as the standard of care [1,2,9,10,11,13,15,18,19].

The aim of this study was to evaluate the impact of simultaneous liver resection and peritoneal cytoreductive surgery with HIPEC on perioperative and survival outcomes. The hypothesis was that this surgical concomitant approach would be associated with higher morbidity and/or mortality than patients with CCR-HIPEC alone.

## 2. Material and Methodology

### 2.1. Study Design and Patient Selection

This is a retrospective analysis of all consecutive patients with concurrent peritoneal and liver metastasis due to colon cancer, treated with peritoneal cytoreduction with HIPEC, in a tertiary referral hospital from January 2008 to October 2021. Two groups of patients were formed. The control group (LR−) consisted of patients who underwent cytoreduction and HIPEC alone, while the experimental group (LR+) consisted of patients who underwent peritoneal cytoreduction with simultaneous resection of liver metastases plus HIPEC. This study was approved by the Multidisciplinary Committee of Peritoneal Surface Oncologic Malignancies and the Investigation Commission of the Universitari Son Espases Hospital.

Inclusion criteria were an Eastern Cooperative Oncology Group (ECOG) score of 0–2, an American Society of Anesthesiologists (ASA) score of 0–3, peritoneal and liver resectable disease, the absence of extra-abdominal metastasis, patients younger than 75 years of age, adequate renal, bone marrow and liver function, and specific written informed consent.

Exclusion criteria were extra-abdominal or unresectable disease, poor performance status (ECOG 3–5 or ASA > 3), progressive disease after neoadjuvant chemotherapy, presence of another neoplasia, and patients having CRS and HIPEC for a Second-Look protocol. Patients with appendiceal or rectal origin were excluded from the analysis. Other exclusion criteria were a PCI higher than 17 points, patients not amenable to complete cytoreduction and lost follow-up.

Data collected. Patient demographics, medical history, and clinical data were collected and analyzed (PCI, number of organs resected, length of operation, grade of cytoreduction, stoma formation, and type of cytostatic and duration of HIPEC). Additionally, perioperative outcomes were included in the analysis (90 days morbidity according to Clavien–Dindo classification, mortality, and transfusion rate), as well as length of intensive care and hospital stay and need for reoperation. In the study, patients who experienced a relapse after undergoing the first CRS-HIPEC procedure and underwent a second CRS-HIPEC procedure were re-enrolled. The calculation of overall survival and disease-free survival (DFS) was performed differently for each procedure. For the first procedure, overall survival was calculated from the date of diagnosis to one day prior to the date of the second surgery and the patient was censored. DFS was calculated from the date of the first surgery to the date of the first recurrence. For the second procedure, overall survival was calculated from the date of the second surgery to the date of death, and DFS was calculated from the date of the second surgery to the date of the second recurrence.

Preoperative Planning. All patients were evaluated by a multidisciplinary committee on peritoneal surface malignancies and liver tumors, made up of surgeons, oncologists, radiologists and nuclear medicine physicians. Radiological evaluation was made with thoracic an abdomen-pelvis CT scan with intravenous contrast and with a positron emission tomography (PET) when indicated by the committee. Patients with liver metastasis were evaluated with hepatic nuclear magnetic resonance. When a high PCI was suspected, laparoscopy was performed to assess the possibility of complete peritoneal cytoreduction in order to avoid unnecessary laparotomies [22]. Neoadjuvant chemotherapy was administered according to the oncologist decision. Preoperative prophylactic intravenous antibiotics (cefotaxime 2 g and metronidazole 500 mg) were infused 30 min before incision and maintained over a 48-h period. Anesthetic strategy was based on general anesthesia, epidural analgesia, invasive monitoring, and goal-directed fluid balance [23].

### 2.2. Follow-Up

A joint follow-up was carried out by oncology and surgery units, with controls one month and three months after the intervention and subsequently every 6 months with clinical examination, CT scan and tumor markers. Postoperative chemotherapy was administered according to the oncologic team.

### 2.3. Operative Technique

A xifopubic incision was routinely made with the patient in the Lloyd Davies position. In patients with liver resection a transversal right flank incision was made as necessary. In all patients with liver metastasis, an intraoperative liver ultrasound was performed. Intraoperatively, volume of peritoneal disease was quantified by the peritoneal carcinomatosis index (PCI) [24] and potential complete cytoreduction was assessed. Liver resections were performed first, followed by peritoneal cytoreduction. Only infiltrated peritoneum by tumor was excised. The decision for resection was established if complete peritoneal cytoreduction and hepatic resection could be achieved. All surgical procedures were performed by experienced surgeons and were standardized to minimize variability. Cytoreductive surgery (CRS) was performed in accordance with techniques described by Bao and Bartlett to achieve CC-0 (no residual macroscopic disease) or CC-1 (residual tumor nodule < 2.5 mm) resection [25]. HIPEC was only performed in cases of optimal peritoneal resection (CC-0 and CC-1) and complete liver resection. A standard institutional protocol for HIPEC was initiated after complete CRS, with the open technique (Coliseum) and target intraperitoneal tissue temperature of 42 °C. We used oxaliplatin in colorectal cancer until 2018, then changed to mitomycin C (20 mg/m^2^ for 60 min and 10 mg/m^2^ for 30 min diluted in 3 L/m^2^ of a 1.5% glucose solution at 42 °C). All safety measures on cytostatic management and control of possible spillages based on the recommendations of this type of procedures were applied [26,27]. Postoperative morbidity was classified according to the Clavien–Dindo grading system [28]. For the purpose of analysis, grades 3–4 were considered major complications. Postoperative morbidity and mortality were registered within 90 days of surgery.

### 2.4. Endpoints

Primary endpoints were postoperative mortality and severe morbidity (Clavien–Dindo grades 3–4) at 90 days. Secondary endpoints were disease free survival (DFS) and overall survival (OS). DFS was defined as the time from CRS-HIPEC to relapse or death. OS was defined as the time from CRS-HIPEC to the time of death due to any cause.

### 2.5. Statistical Analysis

Data are presented as the mean and standard deviation (with a 95% confidence interval), median and interquartile range, or as a percentage (%). To analyze the risks on clinical results, simple and multivariate regression techniques have been applied with the aim of eliminating possible confounding factors and estimating the adjusted effects. For immediate dichotomous results, logistic regressions have been applied and, for numerical ones, linear regressions. Clinical results dependent on follow-up time have been analyzed using COX regression. For immediate dichotomous results, logistic regressions have been applied and, for numerical ones, linear regressions. A value of *p* < 0.05 has been considered as an indicator of a significant difference. The statistical analysis has been developed by the Methodological and Statistical Support Platform of the Balearic Islands Health Research Institute. The statistical software used to analyze the data was IBM-SPSS v.26.

### 2.6. Financial Support

This research was coordinated by ProA Capital, Halekulani S.L., MJR. It was co-financed by the European Development Regional Fund, ‘A way to achieve Europe’, as well as P2022/BMD-7321 (Community of Madrid, Spain).

## 3. Results

### 3.1. Demographics and Perioperative Characteristics

**Figure 1:**
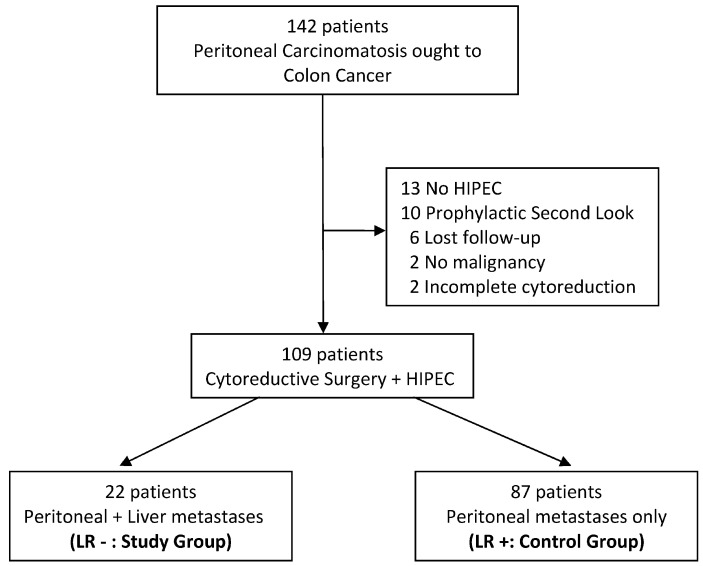
Study flow diagram showing patients entering in the study.

Between January 2010 and October 2022, 142 consecutive patients diagnosed with peritoneal carcinomatosis due to colon cancer underwent cytoreductive surgery and HIPEC. Of these, 33 patients were excluded for different reasons (Figure 1).

Twenty-two patients were included in the liver resection group (LR+: experimental group) and 87 patients were assigned to non-liver resection group (LR−: control group). The demographics of the study population are presented in Table 1. The LR+ group received preoperative systemic chemotherapy more frequently (40.2% vs. 54%), but without significant differences (*p* = 0.226). Variables related to surgical complexity such as PCI, operating time, organs removed, number of anastomoses, and the need for transfusion did not present significant differences. In both groups, the degree of surgical cytoreduction achieved was similar. Table 2 presents the intraoperative and histological characteristics of the liver metastases that were surgically treated. Among patients with liver metastases, the median number and size was 2 cm and 1.7, respectively, and most of them were intraparenchymal. Regarding the surgical technique, splenectomy, lateral resection of duodenum, adrenalectomy and nephrectomy were performed more frequently in LR+ (Table 3).

### 3.2. Morbidity and Mortality

The overall rate of complications was higher in the LR+ group (*p*: 0.024). The LR+ group also had more severe complications (Clavien–Dindo grades III–IV) (54.5% vs. 19.5%; *p*: 0.017). The distribution by type of complications was mostly similar, however, the LR+ group presented a higher incidence of abdominal abscesses (36.4% vs. 14.9%; *p*: 0.034). Although the incidence of postoperative pneumonia was similar, respiratory distress was significantly higher in the LR+ group (36.4% vs. 4.6%; *p*< 0.001). The second more frequent complication was intraabdominal abscesses; it is worth noting that of these infections, five of the eight (62.5%) corresponded to abscesses in the hepatectomy bed. There were no differences in the reoperation rate (Table 4). Univariate analysis showed age, transfusion, and surgical time as predictors of severe complications. However, resection of liver metastases and perioperative transfusion were the only predictor factors in multivariate analysis (Table 5). Three deaths were recorded in the overall series (2.7%), all of them belonging to the LR− group. The causes of death were respiratory failure secondary to bilateral nosocomial pneumonia with respiratory distress in two patients and one haemophagocytic syndrome with massive hemoperitoneum, most likely associated to intraperitoneal oxaliplatin. As happened with intensive care stay, hospital stay was significantly longer in the LR+ group (16 vs. 11 days; *p*: 0.035). A multivariate analysis of predisposing factors for postoperative mortality could not be performed due to the small number of patients.

### 3.3. Survival and Recurrence

**Figure 2:**
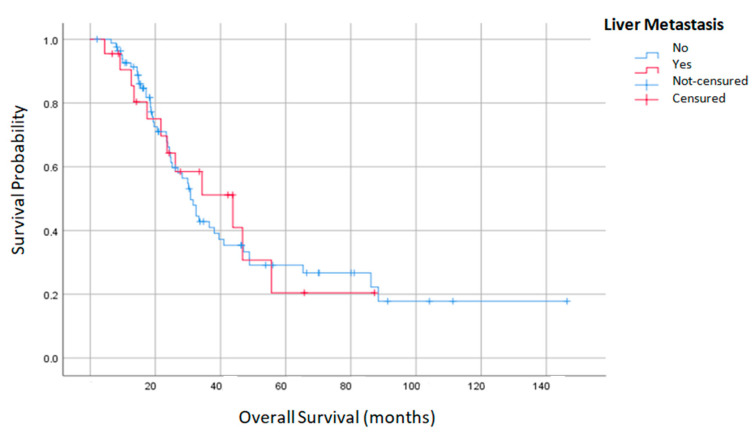
Overall survival of liver plus peritoneal metastases and peritoneal metastasis alone.

**Figure 3:**
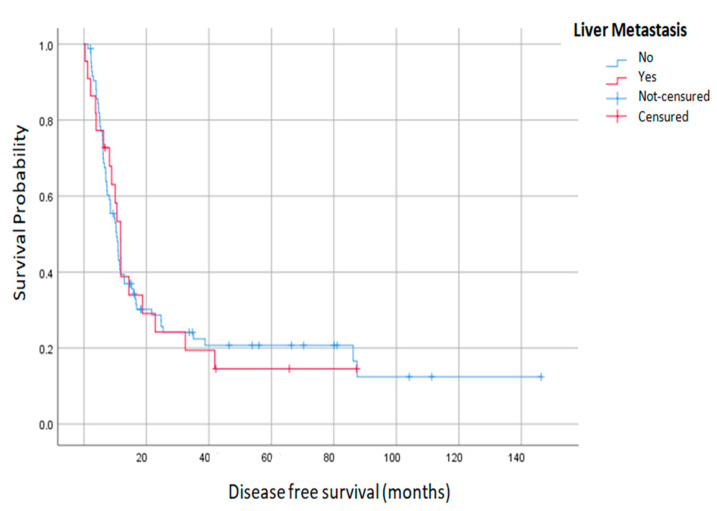
Disease free survival of liver plus peritoneal metastases and peritoneal metastasis alone.

Throughout the study period, a total of 82 patients (75.2%) presented some type of recurrence, with no differences between the two groups. The LR+ group presented a significantly higher liver recurrence (27.8 vs. 10.9%; *p*: 0.049). Median overall survival and DFS of the entire group was 32.4 ± 2.226 and 10.4 ± 0.966 months, respectively. Both groups had no significant differences in overall and DFS survival. The LR+ group registered a higher overall survival (43.8 vs. 30.8 months) (Table 6). Survival at one, three and five years was also similar in the two groups. Only the PCI was shown to be a predictor of overall survival (Figure 2 and Figure 3).

Predictors factors of DFS were PCI and neoadjuvant chemotherapy (Table 7). The recurrence was similar in both groups (64 patients, 73.6% in LR− group and 18 patients, 81.8% in LR+ group) (*p*: 0.43). All recurrence sites were similar in both groups, except for liver recurrence, which was significantly higher in the LR+ group (five patients, 27.8% in LR+ group and seven patients, 10.9% in LR+ group) (*p*: 0.049).

## 4. Discussion

Peritoneal and liver metastases are the two most frequent causes of death in colorectal cancer [10]. In addition, peritoneal dissemination is the one with the worst prognosis with a 30% lower survival, probably due to a lower response to systemic chemotherapy [4,29], or as recently described, by the possibility of representing a mesenchymal molecular subtype (CMS4) with a strong TGF-activation, immune suppression and stromal invasion [30]. Furthermore, it is estimated that approximately 8% of patients with colon cancer develop hepatic and peritoneal metastases simultaneously [1,7] and until recently, these patients were considered unresectable and only amenable to palliative adjuvant chemotherapy with an overall survival of 12 months [4,7]. Indeed, the De Cuba meta-analysis reflected that as much as 25% of scheduled patients for liver surgery were discarded due to the finding of peritoneal metastases [31]. Elias et al. and a consensus statement has shown that patients with up to three liver metastases and a low peritoneal tumor burden (PCI < 12) did not suppose an absolute contraindication for a simultaneous treatment with CRC-HIPEC [9,10,32,33]. Following these criteria and after obtaining a complete tumor resection, a median OS of 25–45 months and an acceptable morbidity and mortality rates could be achieved [1,16,32,34]. In a previous report, we updated our results and showed a median OS of 44 months in patients with simultaneous peritoneal and liver metastases resection [17].

### 4.1. Morbidity and Mortality

Although several studies have reported acceptable results in selected patients treated with simultaneous resection, its general application still remains controversial due to the increased morbidity, mortality, and delayed administration of adjuvant chemotherapy [1,2,9,10,11,12,13,14,15,16,17,18,19,22,33,34]. One added difficulty for this simultaneous approach is the different intraoperative management (restriction of intravenous fluids required during liver resection versus an increased volume perfusion administered during peritoneal cytoreduction and HIPEC) [1]. In this sense, actual goal-guided fluid therapy has helped to treat this problem [23]. The results of this study showed that patients in the LR+ group presented more postoperative complications and longer ICU and hospital stays, despite having a PCI less than 12 and ≤3 liver metastases, which fulfils the criteria proposed by Elias and other authors [9,10,11]. Severe Clavien–Dindo complications were 19.5% vs. 40.5% in LR− and LR+, respectively (*p*: 0.017), and these results are consistent with those found in the literature, which reflects the greater complexity of these interventions, with longer operative time and higher PCI ranging from 15 to 50% [9,11,16,20,21,31,35,36]. In fact, liver metastases and perioperative transfusion were predictors of serious complications in the multivariate analysis (Table 5). Maggiori et al. [34] demonstrated greater postoperative morbidity only in patients with a PCI > 12 who underwent major hepatectomy, considering this association as a limitation factor for the simultaneous approach. Interestingly, this same author and Navez et al. [14] did not related such morbidity to the liver resection. Other authors like Saxena et al. [37] and El-Nakeep et al. [35], did not find significant differences in severe morbidity. The most frequent serious complication in our study was nosocomial pneumonia (23 patients). Eight patients developed respiratory distress and two of them died for this reason. These results force us to insist on activating preventive measures with preoperative and postoperative respiratory physiotherapy, as well as promoting early extubation and mobilization [38]. Intraabdominal abscesses were also significantly more frequent in the LR+ group and this finding may be explained by the fact that in as many as 62.5% of the cases, the infected collection was in the hepatectomy bed. This has been also described by other authors [1]. Unlike severe morbidity, ninety-days mortality has experienced a significant decrease, with figures around 4%, due to better patient selection and postoperative management [1,2,11,39] Our results are in that range and did not show significant differences between the two groups.

### 4.2. Survival and Recurrence

Recently, OS has been increased with the administration of oxaliplatin and irinotecan and the addition of targeted therapies (e.g., bevacizumab and cetuximab) in patients with metastatic colon cancer [40]. However, the survival analysis has biases that are difficult to avoid in patients who are candidates for simultaneous liver and peritoneal cytoreduction surgery because there are no randomized studies that compare the survival obtained with chemotherapy alone [31,34,41]. Although the PRODIGE 7 trial raised questions regarding the efficacy of HIPEC with oxaliplatine [42] in peritoneal metastases, such long-term outcomes (median survival of 42 months and 5-year survival of 40%) have never been published before [42,43]. Our results in the LR+ group (OS and DFS of 43 and 11.7 months, respectively,) are in accordance with those described in previous publications, which range from 15 to 47 months in OS and 8.5 to 25 in DFS, in selected patients (PCI < 12, ≤3 liver metastasis and complete cytoreduction) with concurrent liver and peritoneal metastasis [2,10,11,16,44]. PCI and CC Score are considered the most important prognostic factors in patients with CP [11,45] and a threshold of 17 PCI points has been described as limit for resectability in peritoneal carcinomatosis of colorectal origin [46]. Although the univariate analysis showed differences in the overall survival for the CC score, duration of surgery, serious complications and transfusion, PCI was the only predictive factor for OS (HR: 1.139 (1.089–1.192) *p* < 0.001). However, PCI and neoadjuvant chemotherapy were also predictive factors for DFS (HR: 1.087 (1.047–1.128) *p* <0.001) and (HR: 1.738 (1.092–2.765) *p*: 0.020). For now, we do not have a clear explanation for this last result. One hypothesis could be that those patients who receive neoadjuvant chemotherapy had a higher tumor burden. Despite the radical nature of the surgery, 75% of the patients had a recurrence during the follow-up period. The recurrence rate was similar in both groups, but as described by other authors, the LR+ group had a significant higher liver recurrence (*p*: 0.049) [11,44].

Limitations of this study are the retrospectively nature, the relatively small sample size of the LR+ group and the strict criteria for patient selection. Another limitation is the use of different intraperitoneal cytostatics, but this heterogeneity reflects the evolution of HIPEC treatment over time.

In conclusion, simultaneous peritoneal and liver resection is associated with increased postoperative morbidity and hospital stay, but with similar postoperative mortality and OS and disease-free survival. These results reflect the evolution of these patients considered inoperable until recently and justify the trend to incorporate this surgical strategy within a multimodal therapeutic plan in highly selected patient.

## Figures and Tables

**Table 1:** Demographics and Perioperative characteristics.

Perioperative Data	LR (−) N: 87	LR (+) N: 22	*p*
Age	61.4 (54.9)	66.6 (60.5–71.3)	**0.030**
Female	42 (48.3%)	10 (45.5%)	0.812
Men	45 (51.7%	12 (54.5%)	
ASA-I	8 (9.2%)	3 (13.6%)	NA
ASA-II	55 (63.2%)	12 (54.5%)	
ASA-III	24 (26.4%)	7 (31.8%)	
ECOG			NA
0	52 (59.8%)	18 (81.8%)	
1	32 (36.8%)	4 (18.2%)	
2	2 (2.3%)	0	
3	1 (1.1%)	0	
Charlson	6 (6–7)	7 (6–8)	**0.035**
Preoperative Chemotherapy	35 (40.2%)	12 (54%)	0.226
KRAS mutation	35 (40.2%)	13 (59.1%)	0.111
Surgical PCI	8 (3–14)	9 (6–14)	0.380
Operative Time (minutes)	467 (390–567)	512 (456–638)	0.117
No. resected organs	4 (2–4)	4 (3–5)	0.171
No. of anastomosis	1 (1–3)	1 (1–3)	0.735
CCR-0	83 (95.4%)	21 (95.4%)	0.581
CCR-1	4 (4.6%)	1 (4.6%)	
Transfusion rate Blood packs/patient	37 (42.5%) 1.4 (0–10)	11 (50%) 2.7 (0–12)	0.528
Stoma formation	4 (4.6%)	0	0.581

**Table 2:** Liver metastases characteristics.

Liver Metastases	n: 22
Location	
Subcapsular	5 (22.7%)
Intraparenchymal	15 (68.2%)
Both	2 (9.1%)
Number of metastases	
1	9 (40.9%)
2	11 (50%)
3	2 (9.1%)
Size (cm)	2
Type of liver resection	
Segmentectomy	9 (40.9%)
Atypical resection	13 (59.1%)

**Table 3:** Types of organ resection.

Types of Resected Organ	LR (−) No (%)	LR (+) No (%)	*p*
Peritoneum	79 (21.9)	17 (19.9)	0.34
Omentectomy	62 (17.4)	10 (11.6)	0.06
Diaphragm resection	8 (2.2)	4 (4.6)	0.20
Gastric resection	4 (1.1)	0	0.31
Cholecystectomy	38 (10.7)	13 (15.1)	0.15
Splenectomy	16 (4.5)	0	**0.03**
Duodenum (lateral resection)	0	3 (3.5)	**0.0004**
Pancreatectomy (corporo-caudal)	3 (0.8)	0	0.38
Adrenalectomy	0	1 (1.2)	**0.004**
Small bowel	33 (9.3)	8 (9.3)	1
Right/Transverse colectomy	30 (8.5)	7 (8.1)	0.91
Left/Sigmoid/Rectal resection	36 (10.1)	10 (11.6)	0.63
Subtotal colectomy	5 (1.4)	0	0.26
Nephrectomy	0	3 (3.5)	**0.0004**
Ureter resection	6 (1.7)	2 (2.3)	0.68
Cystectomy	2 (1.1)	1 (1.2)	0.54
Hysterectomy/Ovarian resection	23 (6.5)	4 (4.6)	0.48
Aortic Lymphadenectomy	10 (2.8)	3 (3.5)	0.82

**Table 4:** Morbidity and Mortality.

Postoperative Complications	LR (−) N: 87	LR (+) N: 22	
Overall Morbidity (90 days)	22 (25.3%)	11 (50%)	**0.024**
Clavien–Dindo (90 days)			
Grade 0	14 (17.7%)	5 (13.5%)	
Grade 1	15 (19%)	2 (5.4%)	0.059
Grade 2	33 (41.8%)	15 (40.5%)	
Grade 3	6 (7.6%)	5 (13.5%)	**0.017**
Grade 4	9 (11.4%)	10 (27%)	
Reinterventions	5 (5.7%)	2 (9.1%)	0.567
-Evisceration	2	0	
-Colonic fistulae	1	0	
-Abdominal abscess	1	0	
-Anastomotic dehiscence	1	1	
-Ileus	0	1	
Overall Mortality (90 days)	3 (2.7%)		
Mortality 90 (days)	3 (3.4%)	0 (0)	1.000
Causes of death			
-Bilateral Pneumonia and distress	2	0	
-Hemophagocytic syndrome	1	0	
ICU length of stay (median)	2 (2–3)	3 (2–4)	**0.039**
Hospital length of stay (median)	11 (9–16)	16 (10–35)	**0.035**
Abdominal abscess	13 (14.9%)	8 (36.4%)	**0.034**
Superficial SSI	10 (11.5%)	4 (8.2%)	0.475
Small bowel fistula	3 (3.4%)	1 (4.5%)	1.000
Anastomotic leak	2 (2.3%)	1 (4.5%)	0.495
Hemoperitoneum	2 (2.3%)	1 (4.5%)	0.495
Chylous ascites	0	1 (4.5%)	0.202
Hemothorax	1 (1.1%)	0	1.000
Thrombocytopenia	19 (21.8%)	9 (40.9%)	0.067
Ileus	11 (12.6%)	6 (27.3%)	0.106
Leukopenia	8 (9.2%)	1 (4.5%)	0.683
Pneumoniae	8 (9.2%)	3 (13.6%)	0.691
Pleural effusion with drainage	4 (4.6%)	2 (9.1%)	0.599
Pneumonia and respiratory distress	4 (4.6%)	8 (36.4%)	**0.001**
Central line sepsis	3 (3.4%)	0	1.000
Urinary infection	3 (3.4%)	1 (4.5%)	1.000
Stroke	2 (2.3%)	1 (4.5%)	0.495
Ulcerative gastritis	2 (2.3%)	0	1.000
Acute pancreatitis	1 (1.1%)	0	1.000

**Table 5:** Predictive factors for Severe Complications (III–IV Clavien–Dindo).

Variables	OR Crude	*p*	OR Adjusted	*p*
Liver metastases	4.29 (1.61–11.46)	**0.004**	4.36 (1.41–13.50)	**0.011**
Transfusion	3.25 (1.36–7.74)	**0.008**	3.36 (1.23–9.21)	**0.019**
Age	1.13 (0.57–2.23)	0.658	0.99 (0.98–1.01)	0.438
ASA	1.13 (0.57–2.23)	0.722	1.14 (0.53–2.44)	0.722
ECOG	0.51 (0.22–1.20)	0.124	0.72 (0.29–1.82)	0.494
PCI	1.02 (0.96–1.09)	0.533	0.99 (0.90–1.08)	0.747
Neoadjuvant chemotherapy	1.35 (0.58–3.11)	0.484	1.14 (0.44–2.96)	0.785
Operative time	1.00 (1.00–1.01)	0.112	1.00 (1.00–1.01)	0.644

**Table 6:** Survival.

Survival	LR (−) N: 87	LR (+) N: 22	*p*
Median Overall Survival	30.8 ± 2.223	43.8 ± 13.373	0.905
1 year	92%	90%	
3 years	37.7%	43.8%	
5 years	21.1%	14.3%	
Median Disease Free Survival	10.5 ± 1.257	11.7 ± 1.297	0.938
1 year	40%	38%	
3 years	22%	14%	
5 years	16%	14%	

**Table 7:** Predictive factors of survival.

Survival Predictive Factors		Univariate Analysis		Multivariate Analysis	
Survival	Variables	HR	*p*	HR Cox Regression	*p*
Overall Survival	-PCI	1.15 (1.10–1.20)	0.000	1.139 (1.089–1.192)	**0.001**
	-Neoadjuvant Chemo	1.93 (1.16–3.21)	0.012		
	-CC score	7.67 (2.25–26.18)	0.001		
	-Operative time	0.48 (0.10–2.34)	0.002		
	-Severe complications (III–IV) *	1.32 (1.05–1.65)	0.016		
	-Transfusion	1.78 (1.07–2.96)	0.0927		
Disease Free Survival	-PCI	1.09 (1.05–1.13)	0.000	1.087 (1.047–1.128)	**0.001**
	-Neoadjuvant Chemo	1.82 (1.18–2.82)	0.007	1.738 (1.092–2.765)	**0.020**
	-No. of liver metastases	25.51 (3.11–209.4)	0.003		
	-Operative time	1.00 (1.00–1.00)	0.029		
	-Severe complications (III–IV) *	25.51 (1.04–1.50)	0.016		
	-Native KRAS	9.70 (1.01–93.23)	0.049		

* III–IV Clavien–Dindo complications.

## Data Availability

The data of the study were extracted from a prospective database in which the anonymity of the patients was sought.
